# *Physalis alkekengi* L. Extract Reduces the Oxidative Stress, Inflammation and Apoptosis in Endothelial Vascular Cells Exposed to Hyperglycemia

**DOI:** 10.3390/molecules25163747

**Published:** 2020-08-17

**Authors:** Laura Gratiela Vicas, Tunde Jurca, Ioana Baldea, Gabriela Adriana Filip, Diana Olteanu, Simona Valeria Clichici, Annamaria Pallag, Eleonora Marian, Otilia Micle, Carmen Bianca Crivii, Tudor Suciu, Izabela Craciun, Felicia Gabriela Gligor, Mariana Muresan

**Affiliations:** 1 Department of Pharmacy, Faculty of Medicine and Pharmacy, University of Oradea, 29 Nicolae Jiga Street, 410028 Oradea, Romania; laura.vicas@gmail.com (L.G.V.); jurcatunde@yahoo.com (T.J.); annamariapallag@gmail.com (A.P.); marian_eleonora@yahoo.com (E.M.); 2 Department of Physiology, “Iuliu Hatieganu” University of Medicine and Pharmacy, 1-3 Clinicilor Street, 400006 Cluj-Napoca, Romania; baldeaioana@gmail.com (I.B.); simonaclichici@yahoo.com (S.V.C.); suciu_ts@yahoo.com (T.S.); 3 Department of Preclinical Disciplines, Faculty of Medicine and Pharmacy, University of Oradea, 10 Piata 1 Decembrie Street, 410073 Oradea, Romania; micleotilia@yahoo.com (O.M.); marianamur2002@yahoo.com (M.M.); 4 Morphology Department, “Iuliu Hatieganu” University of Medicine and Pharmacy, 3-5 Clinicilor Street, 400006 Cluj-Napoca, Romania; bianca.crivii@umfcluj.ro; 5 Faculty of Medicine, Lucian Blaga University Sibiu, Lucian Blaga Street No 2A, 550169 Sibiu, Romania; isabel1noel@yahoo.com (I.C.); feligligor@yahoo.fr (F.G.G.)

**Keywords:** hyperglycemia, oxidative stress, inflammation, apoptosis, polyphenols, Physalis extract

## Abstract

To find new natural remedies in diabetes, this study investigated the biological activity of two extracts obtained from the fruits (PhyF) and herba (PhyH) of *Physalis alkekengi var. franchetii* L. on human umbilical vein endothelial cells (HUVECs) exposed to normo- and hyperglycemic conditions. The biological effect was quantified by malondialdehyde, IL-31 and IL-33 levels in correlation with physico-chemical characterization and antioxidant activity. Additionally, from PhyP extract, the caspase-3, IL-6, IL-10, tumor necrosis factor (TNF)-α and nuclear transcription factor NFkB expressions were evaluated. HPLC analysis revealed a significant number of phenolic compounds, especially in PhyF extract, with a good antioxidant activity as highlighted by TEAC, CUPRAC or DPPH methods. On HUVECS cells, the extracts were not toxic even at high concentrations. Particularly PhyF extract, diminished lipid peroxidation and inhibited the IL-31 and IL-33 secretions induced by hyperglycemia. The inhibitory effect on proinflammatory cytokines was noticed after both doses of PhyF extract in parallel with the upregulation of anti-inflammatory cytokine IL-10. Moreover, PhyF, especially in a low dose, reduced caspase-3 active form. These experimental findings suggest that Physalis fruits extract exerted beneficial effects in hyperglycemia by inhibition of oxidative stress, inflammation and apoptosis being a good adjuvant option in diabetes.

## 1. Introduction

According to the World Health Organization, diabetes mellitus (DZ) is one of the four major non-communicable diseases and the third risk factor for premature mortality [1]. Diabetes is induced by the disorder of pancreatic insulin secretion or by the resistance of peripheral cells to the insulin action. The results of impaired insulin effect are hyperglycemia and imbalance of metabolism, which lead to hyperlipidemia, inflammation and consequently oxidative stress [2,3]. Moreover, the high levels of blood glucose during a lengthy and insufficient treatment cause various complications related to endothelial dysfunction such as retinopathy, kidney failure, neuropathies and macrovascular disease [4].

In the increased incidence of type 2 DZ, a central role is played by obesity due to overeating and a sedentary lifestyle. Besides lipotoxicity [5,6], local inflammation also occurs, with the secretion of TNF-α by adipocytes and macrophages attracted to adipose tissue and consequently insulin resistance [7].

In DZ, the main source of oxidative stress is mitochondria and mitochondrial electron transport chain overload [8]. In addition, the normal intermediates of glycolysis such as fructose-1.6-bisphosphate and glyceraldehyde-3-phosphate will follow other alternative pathways and generate reactive oxygen species (ROS) [9]. It will also determine non-enzymatic glycosylation of protein fractions and activation of the hexosamine pathway, the polyol pathway and the protein kinase C (PKC) system [10]. As a result of PKC activation, the overexpression of endothelial vascular growth factor (VEGF), plasminogen activator inhibitor (PAI)-1, nuclear kappa B (NFkB) transcription factor and transforming growth factor (TGF)-β and ROS generation occur [11]. Hyperglycemia and oxidative stress also increase DNA damage, lipid peroxidation and protein oxidation and inactivate the antioxidant enzyme system.

A high level of glucose activates the synthesis of TGF-α and PAI-1, promoting microvascular lesions [10]. Furthermore, in hyperglycemia, NADPH consumption in the polyol pathway enhances intracellular oxidative stress, affecting vascular endothelium [9,12]. In addition, apoptosis and vascular permeability due to glucotoxicity induce a chronic inflammatory process by activation of NFkB in the endothelial cells [13,14,15,16].

Apoptosis is stimulated by external or internal events, the most known extrinsic stimulus being the death receptor including Fas and TNFR1 (Tumor Necrosis Factor Receptor-1), which in turn may promote the pro-inflammatory pathways leading to the activation of NFkB [17]. The activation of nuclear transcription factor NFkB induces a chronic inflammatory status by increasing the synthesis of adhesion molecules and proinflammatory cytokines, including TNF-α and interleukin (IL)-1, factors responsible for increasing the permeability of endothelial cells [13,14,18,19].

The endothelial microlesions are aggravated by the imbalance between vasodilation (NO and prostacyclin) and vasoconstrictive agents such as endothelin and thromboxane A_2_ [13]. Both prostacyclin and thromboxane are arachidonic acid derivatives produced by cyclooxygenase, an enzyme which in turn increases the production of ROS and activates the NFkB pathway [12].

Interleukin-33 is part of the IL-1 superfamily of cytokines and it is expressed in epithelial and endothelial cells, both in inflammation and in normal cell homeostasis. IL-33 can function equally as a cytokine and nuclear factor that regulates the gene transcription. In the absence of inflammation, IL-33 is located in the nucleus where it acts as a nuclear factor with a role in transcription [20]. It seems that IL-33 sequesters nuclear NFkB and thus reduces the activation of inflammatory pathways triggered by NFkB [21]. At the same time, IL-33 can function as an “alarm” released after cell necrosis or inflammation to alert the immune system to tissue damage or stress. Following the interaction with ST2 receptors (IL-1RL1) and the accessory protein of IL-1 receptor (IL-1RAcP), IL-33 induces the activation of NFkB and mitogen-activated protein kinase (MAPK) and increases the secretion of Th2 cytokines [22]. A recent study reported an increase of IL-33 and ST2 expressions in adipocytes and adipose tissues [23] as well as in endothelial and epithelial cells in obesity. In addition, the IL-33/ST2 axis might influence the generation of Th17 producing IL-31 and consequently the progression of inflammatory diseases. IL-31 is involved in the release of proinflammatory cytokines, by activation of three signaling pathways: JAK/STAT, PI3K-AKT and MAPK pathways [24].

IL-6 is known as a major regulator of acute-phase inflammatory response with an important role in the initiation of chronic inflammation and the promotion of micro- and macrovascular lesions in diabetes. It contributes to endothelial dysfunction by the stimulation of monocyte chemotactic protein-1 and cell adhesion molecules such as intercellular adhesion molecule (ICAM) 1 and vascular cell adhesion molecule (VCAM) 1 [25]. The synthesis of IL-6 and TNF-α is inhibited by IL-10, an anti-inflammatory cytokine secreted by monocytes/macrophages [26], involved in nuclear translocation of the signal transducer and the activator of transcription (STAT)-3 [27]. IL-10 increases the glucose uptake, improves the insulin sensitivity and inhibits the TNF-α mediated insulin resistance in adipocytes and skeletal muscle [28] with lowering glycaemia.

Experimental data show that some natural plant compounds can indirectly provide protection by an antioxidant effect, modulate the signaling pathways involved in cell proliferation and cell cycle progression or inhibit inflammation and promote the apoptosis of damaged cells.

Polyphenols found in spices, vegetables and fruits can also reduce the activity of prooxidative NADPH oxidases and stimulate antioxidative enzymes and eNOS [29,30], providing protection against oxidative stress.

*Physalis alkekengi* L., bladder cherry, Chinese lantern plant, is a perennial herbaceous plant which grows naturally in the regions covering Southern Europe and South and Northeast Asia [31,32]. The taxonomic classification of *Physalis alkekengi* L. has shown that it is a part of the *Solanaceae* family, *Solanales* order, *Magnoliopsida* class, *Magnoliophyte phylum*. *Physalis alkekengi* L. samples used in this study, originating from the planted culture of Bihor County, were carefully selected from unpolluted areas, in 2018. The fruit of *Physalis alkekengi* L is a round cherry-sized shiny red berry, which is hidden in a scarlet red papery fruit covering, which is grown from the calyx. *Physalis alkekengi* L. is a valuable resource for phytochemicals used for medicinal purposes [31,33]. The phytochemical investigations indicate that *Alkekengi fructus* contains lycopene, ascorbic acid, alkaloids and glucocorticoids, and it is a rich source of polyphenols, with an antioxidant effect [34,35]. It has been demonstrated that *Alkekengi fructus* has anti-inflammatory, antibacterial, antimycotic, anti-gout, analgesic, diuretic and laxative effects. Numerous other studies have shown radical scavenger and lipid peroxidation inhibitory activities [36,37,38]. In fact, the plant has ethnopharmacological relevance being its calyxes and fruits mentioned in traditional Chinese medicine. Some researchers have highlighted the pharmacological effects in the treatment of various diseases, including cough, pharyngitis, eczema, etc. [39].

Based on these data, the study aimed to evaluate the physico-chemical characterization of two extracts obtained from fruits and herba of *Physalis alkekengi var. franchetii* L. as well as their antioxidant abilities in vitro to assess their biological activities. The effect of extracts was quantified on human endothelial cells HUVECs exposed to normo- and hyperglycemic conditions by oxidative stress, proinflammatory and anti-inflammatory cytokines, caspase-3 active form and transcription factors expressions.

## 2. Results

### 2.1. The Polyphenolic Profiles of Two Physalis Extracts

To identify and quantify the bioactive compounds of the two extracts, HPLC-RP with UV detection was used. HPLC analysis with a diode array detector revealed a significant number of phenolic compounds, especially in Physalis fruits. The results indicate a high concentration of gallic acid (32.697 ± 0.97 mg/kg) and rutin (14.505 ± 0.32 mg/kg) in fruits compared to the herba (Table 1). Resveratrol (22.626 ± 0.31 mg/kg) was found only in the Physalis fruits. We noticed the absence of resveratrol, catechin and chlorogenic acid in the Physalis herba. The respective retention times, λ max, are shown in Table 1 for bioactive compounds identified in Physalis fruits and herba.

Due to the structural diversity of the phenolic compounds (polyphenols and flavonoids), we also determined the total polyphenolic and total flavonoids content of the two extracts. The results are shown in Table 2. A plant tissue dependent variation was found for both types of phenolic compounds investigated. Thus, the total polyphenols were increased in significant concentrations in fruits; the extract of Physalis fruits was richer in polyphenols (1082.5 ± 1.98 mg GAE/100 g DW) than the extract of Physalis herba (221.6 ± 1.68 mg GAE/100 g DW). The flavonoids contents found in the two different samples of the analyzed Physalis are presented in Table 2. A tissue-dependent variation was also found. Thus, the content of herbal flavonoids was 0.297 ± 0.53 mg QE/100 g DW while the same parameter in the fruits was 0.695 ± 0.53 mg QE/100 g DW. The concentration of flavonoids was significantly increased in the Physalis fruits. The results are included in Table 2.

### 2.2. The Antioxidant Activity of the Two Physalis Extracts

Both studied extracts showed a good antioxidant activity. Thus, Physalis fruits extract had a free radical capture capacity evaluated by the DPPH method of 75.00 ± 0.29% compared to 30.45 ± 0.31% of Physalis herba. This increased antioxidant activity of the Physalis fruits extract was also demonstrated by ionic mechanisms, when it acted as reducing agents in the FRAP and CUPRAC methods (Table 2). The three methods (DPPH, FRAP and CUPRAC) used for the evaluation of antioxidant activity showed different antioxidant potentials depending on the type of extract tested. The results show that *Physalis alkekengi* from the plant culture of Bihor County had a content of polyphenols and flavonoids, as well as an antioxidant capacity, similar to those obtained by other research groups [38,40,41].

### 2.3. Cell Viability

**Figure 1:**
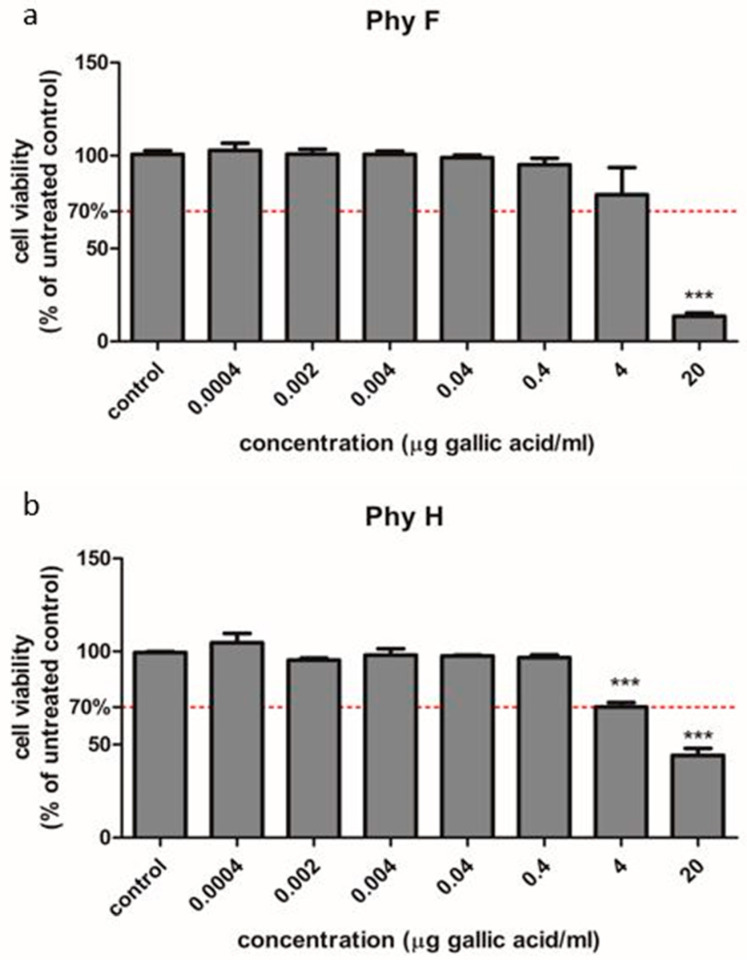
Cell viability of HUVECs cells treated with Physalis herba and fruit extracts in different concentrations compared to untreated cells. To evaluate the viability, HUVECs cells were exposed to PhyF (**a**) and PhyH (**b**) extracts in concentrations ranging from 0.0004 to 20 µg GAE/mL. The viability was tested through the colorimetric measurement of formazan using CellTiter 96^®^ AQueous Non-Radioactive Cell Proliferation Assay. Data are presented as percent of untreated controls (mean ± SD, *n* = 3 for each sample). *** *p* < 0.001.

To evaluate the efficacy of Physalis herba and Physalis fruits in hyperglycemic conditions, HUVECs exposed to normo- and hyperglycemia were used. The effects of the two compounds were evaluated by a viability test, oxidative stress and inflammatory markers assessment including IL-31 and IL-33 levels. Additionally, for PhyF, TNF-α level and caspase-3 active form in supernates and the expressions of NFkB, pNFkB, IL-6 and IL-10 in cell lysates were assessed. The cell viability of HUVECs was evaluated with different concentrations of the two extracts (Figure 1a,b). The dose that decreased the viability of the treated cells below 70% was considered toxic. In HUVEC cells treated with either PhyF or PhyH extracts, the viability was not modified at concentrations ≤0.4 µg GAE/mL. At higher concentrations, the viability of the cells was decreased, depending on the concentration, bellow the toxicity limit of 70%. This shows that the extracts are well tolerated at doses up to 0.4 µg GAE/mL, without signs of cytotoxicity. Therefore, the following experiments were done using doses of 0.1 and 0.05 µg GAE/mL for both extracts.

### 2.4. The Influence of Two Extracts on Oxidative Stress in HUVECs

**Figure 2:**
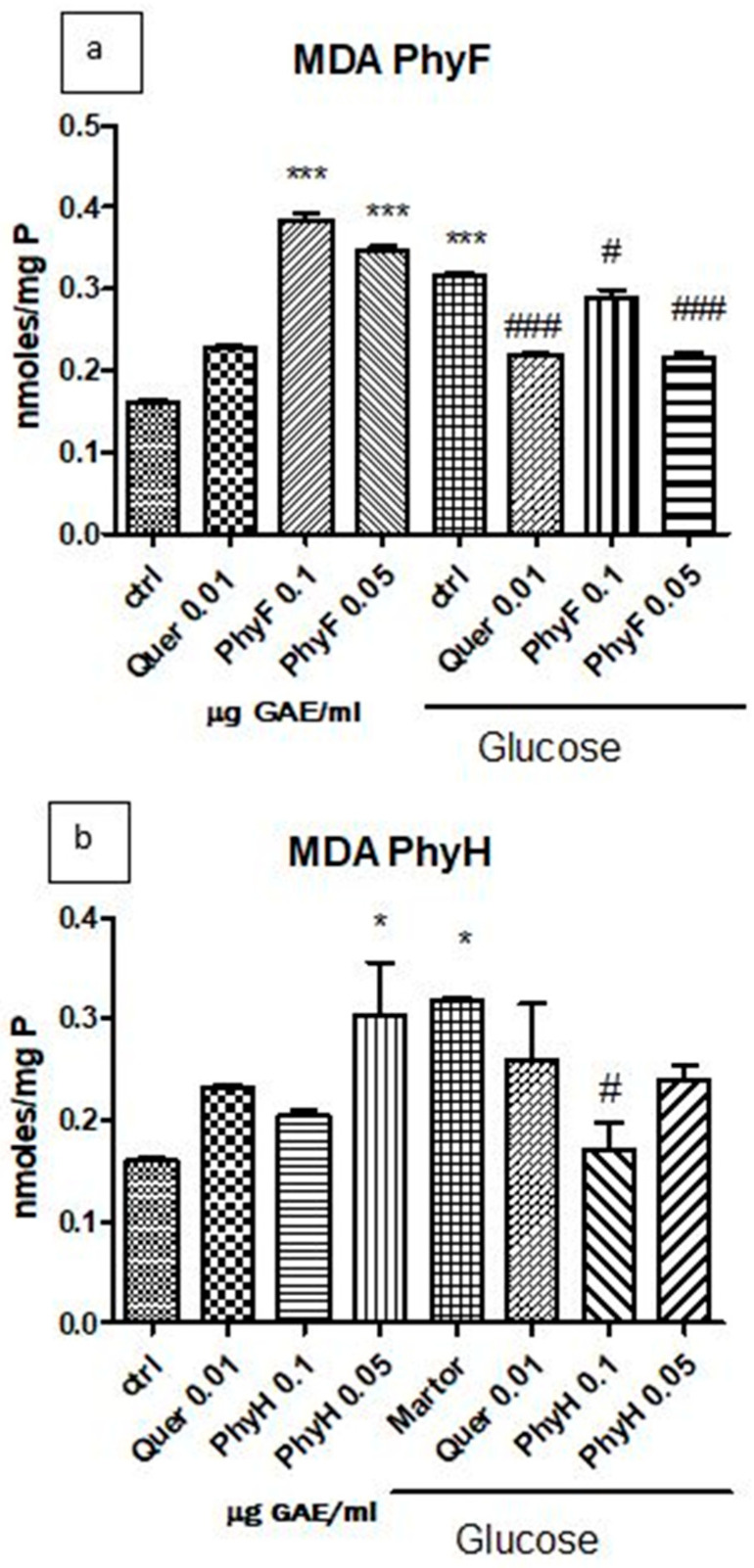
Malondialdehyde levels in HUVECs lysates exposed to hyperglycemia and normoglycemia and treated with Physalis extracts and quercetin. (**a**) The MDA levels (nmoles/mg protein) after exposure to hyperglycemia and pretreatment for 24 h with Physalis fruits extract (0.05 and 0.1 μg GAE/mL) and quercetin. (**b**) MDA levels (nmoles/mg protein) after exposure to hyperglycemia and PhyH extract and quercetin. The statistical significance of the difference between treated and control groups was evaluated with one-way ANOVA, followed by Tukey’s Multiple test. * *p* < 0.05; *** *p* < 0.001, treated vs. untreated cells in normoglycemia; and control in hyperglycemia vs. control in normoglycemia; ^#^ *p* < 0.05, ^###^ *p* < 0.001, treated groups in hyperglycemia vs. control group in hyperglycemia.

Hyperglycemia has a pro-oxidant effect on the membrane lipids of endothelial cells, as displayed by the increased malondialdehyde level (*p* < 0.0001) compared to the control, untreated cells in normoglycemia (Figure 2a,b). The two doses of Physalis fruits extract increased malondialdehyde levels in cells exposed to normoglycemia, while, in hyperglycemia, the two extracts diminished lipid peroxidation at both doses (*p* < 0.05 and *p* < 0.001). The low dose of PhyF had a better antioxidant effect compared to the high dose, the effect being close to that obtained with quercetin (*p* < 0.001). Physalis herba was prooxidant in normoglycemia only at a low dose, while, in cells exposed to hyperglycemia, it exerted an antioxidant effect only at a high dose (*p* < 0.05). The low dose of PhyH extract did not influence the lipid peroxidation in hyperglycemic conditions (*p* > 0.05). The best protection of endothelial cells against hyperglycemia was exerted by the low concentration of the PhyF extract, the high dose of the PhyH extract and quercetin (*p* < 0.001; *p* < 0.05 and *p* < 0.001).

### 2.5. The Influence of the Physalis Extracts on NFkB Activation and Inflammatory Markers in HUVECs

**Figure 3:**
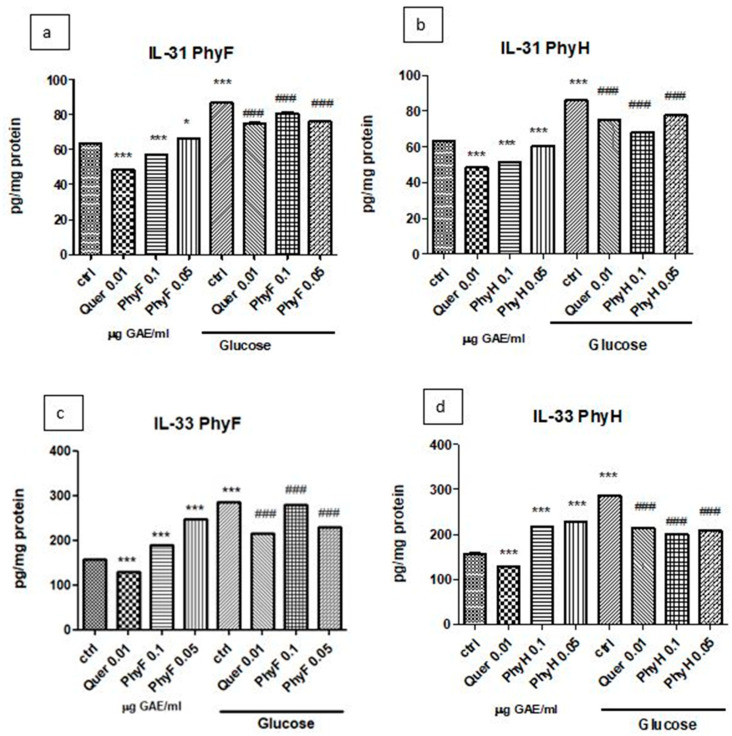
IL-31 and IL-33 levels in HUVECs cells exposed to normo- and hyperglycemia and treated with Physalis extracts and quercetin. Inflammatory markers in cell lysates of HUVECs cells exposed to Physalis extracts (0.05 and 0.1 μg GAE/mL PhyF and PhyH) and quercetin. Protein levels of IL-31 (**a**,**c**) and IL-33 (**b**,**d**) were determined by ELISA (pg/mg protein). Each bar represents a mean ± standard deviation (*n* = 3). The statistical significance of the difference between treated and control groups was evaluated with one-way ANOVA, followed by Tukey’s Multiple test, * *p* > 0.05; *** *p* < 0.001, treated vs. untreated cells in normoglycemia and control in hyperglycemia vs. control in normoglycemia; ^###^ *p* < 0.001, treated groups in hyperglycemia vs. control group in hyperglycemia.

The exposure of HUVECs cells to hyperglycemia induced IL-31 secretion compared to normoglycemia (*p* < 0.001). The administration of quercetin and the high dose of PhyF extract to cells exposed to normoglycemia significantly reduced IL-31 levels (*p* < 0.001) compared to the control, untreated cells (Figure 3a,b). The same pattern was observed when the two doses of PhyH to cells in the same medium (*p* < 0.001) were administered. The pretreatment of HUVECs exposed to hyperglycemia with two doses of PhyF and quercetin for 24 h significantly diminished the IL-31 secretion (*p* < 0.001). A similar effect was noticed in the incubation of cells with two doses of PhyH extract in hyperglycemia (*p* < 0.001). IL-33 secretion increased in cells exposed to hyperglycemia medium compared to cells in normoglycemia (*p* < 0.001) (Figure 3c,d). In normoglycemia, quercetin administration diminished the IL-33 levels, while the two extracts, in both doses, significantly increased IL-33 (*p* < 0.001). This pattern was also found in the hyperglycemia condition in the case of the quercetin administration. In hyperglycemia, the inhibitory effect on IL-33 secretion was noticed after a low dose of PhyF extract and after both doses of PhyH.

**Figure 4:**
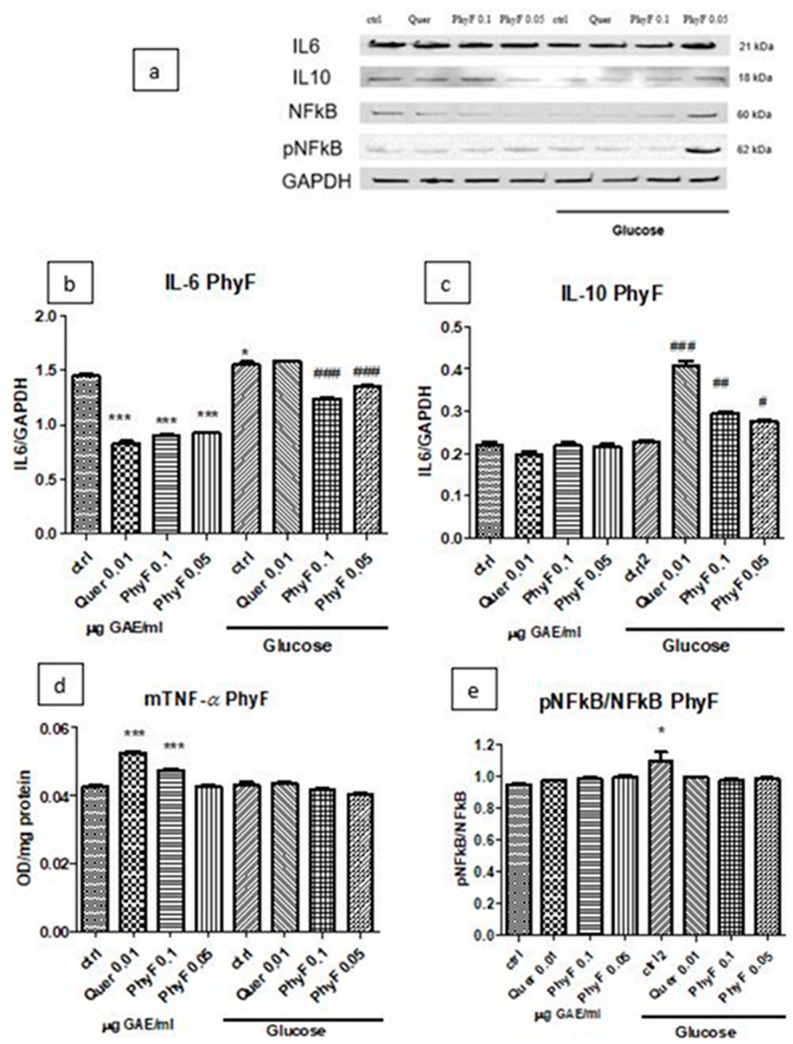
TNF-α, IL-6 and IL-10 levels and pNFkB/pNFkB ratio in HUVECs cells exposed to normo- and hyperglycemia and treated with Physalis fruits extract and quercetin. Representative images of immunoblotting for IL-6, IL-10, NFkB, pNFkB and GAPDH in cell lysates of HUVECs exposed to 0.05 and 0.1 μg GAE/mL Physalis fruits extract (PhyF) and quercetin are shown in the upper panel (**a**) and the results of statistical analysis for the ratio of IL-6, IL-10 and GAPDH expression in the lower panels (**b**,**c**). Image analysis of two bands, NFkB and pNFkB, were completed by densitometry (**e**) and the results are expressed as pNFkB divided by the total NFkB. (**d**) TNF-α levels were determined by ELISA (OD/mg protein). Each bar represents mean ± standard deviation (*n* = 3). The statistical significance of the difference between treated and control groups was evaluated with one-way ANOVA, followed by Tukey’s Multiple test. * *p* < 0.05; *** *p* < 0.001, treated vs. untreated cells in normoglycemia and control in hyperglycemia vs control in normoglycemia respectively; ^#^ *p* < 0.05, ^##^ *p* < 0.01; ^###^ *p* < 0.001, treated groups in hyperglycemia vs. the control group in hyperglycemia.

Based on the results obtained on the viability test and the evaluation of oxidative stress, IL-31 and IL-33 levels, PhyF was chosen for further evaluations. IL-6 and IL-10 expressions after treatment with quercetin and PhyF extract were quantified in cell lysates by Western blot (Figure 4a–c). Hyperglycemia induced a proinflammatory status, an effect proved by the increase of IL-6 secretion (*p* < 0.05). The quercetin administration or treatment with both doses of PhyF extract reduced the IL-6 levels in normoglycemia (*p* < 0.001), while, in hyperglycemia, the protective effect was exerted only by 0.1 and 0.05 µg GAE/mL PhyF extract (*p* < 0.001). IL-10, an anti-inflammatory cytokine, increased in hyperglycemic conditions in cells treated with quercetin and the two doses of PhyF extract, suggesting the protective effect of tested substances. The best protection was exerted by quercetin (*p* < 0.001) and the high dose of PhyF extract (*p* > 0.01). The TNF-α secretion, assessed in the medium in normoglycemic conditions, increased after quercetin (*p* < 0.001) and PhyF (*p* < 0.001) administrations. (Figure 4d). The untreated cells exposed to hyperglycemia or hyperglycemia associated with quercetin or Physalis fruits extract showed the same levels of TNF-α without a statistical significance between the groups. The effect of PhyF exposure on NFkB activation was quantified by the expression of constitutive and phosphorylated forms of NFkB in HUVECs lysates (Figure 4a,e). The active form from the total form of NFkB was assessed comparatively in cells exposed to hyperglycemia and normoglycemia and also after quercetin or two doses of PhyF extract administrations. The hyperglycemic condition induced the activation of NFkB in correlation with the increase of lipid peroxidation (*p* < 0.05) compared to normoglycemia. Quercetin or PhyF administrations did not influence the activation of NFkB (*p* > 0.05).

### 2.6. The Influence of the Physalis Puree Extract on Apoptosis

**Figure 5:**
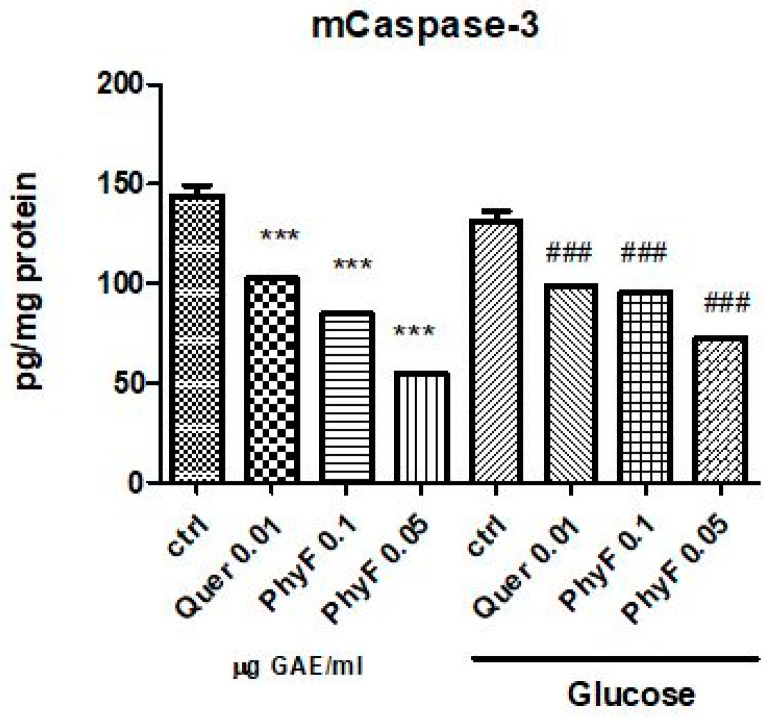
Caspase-3 active in HUVECs cells exposed to normo- and hyperglycemia and treated with Physalis fruits extract and quercetin. Caspase-3 active from supernates of cell treated with 0.05 and 0.01 μg GAE/mL and quercetin were evaluated by ELISA (pg/mg protein). Each bar represents a mean ± standard deviation (*n* = 3). The statistical significance of the difference between treated and control groups was evaluated with one-way ANOVA, followed by Tukey’s Multiple test, *** *p* < 0.001, treated vs. untreated cells in normoglycemia and control in hyperglycemia vs control in normoglycemia; ^###^ *p* < 0.001, treated groups in hyperglycemia vs. control group in hyperglycemia.

In normoglycemia, the administration of quercetin and two doses of extract diminished the caspase-3 active form (*p* < 0.001) (Figure 5). The same effect had the treatment with a low and high dose of PhyF, demonstrating the antiapoptotic effect of the PhyF extract. Quercetin reduced the caspase-3 active in medium compared to the control, untreated cells (*p* < 0.001).

## 3. Discussion

Endothelial dysfunction in diabetes is the initiating factor of micro- and macrovascular complications and is due to the disruption of balance between vasodilatation and vasoconstriction with the inhibition of nitric oxide synthesis and generation of free radicals, vascular inflammation, increased vascular permeability and insulin resistance [42,43]. The understanding of endothelial dysfunction pathogenesis and the response of endothelial cells to different drugs is crucial for vascular complications prevention. In recent years, new treatments have been developed for hyperglycemia, but these agents sometimes have side effects or are more difficult to tolerate in a long-term use. Therefore, the use of natural agents, rich in bioactive compounds, without demonstrated side effects, is a good strategy to maintain the glucose homeostasis and prevent the occurrence of disease.

Our study demonstrated that exposure to hyperglycemic medium induced oxidative stress, inflammation and slight apoptosis, compared to normoglycemia. These effects were attenuated by plant and fruits extracts, particularly by Physalis fruits extract. Thus, the two doses of Physalis fruits extract and the high dose of Physalis herba were non-toxic, and they diminished lipid peroxidation and reduced IL-31 and IL-33 secretions. Moreover, both doses of Physalis fruits extract decreased IL-6 levels and increased IL-10 in hyperglycemia in parallel with a reduction of caspase-3. These effects were in correlation with an increased content of flavonoids and high antioxidant activity of Physalis fruits extract, as demonstrated by DPPH, FRAP and CUPRAC methods.

The results are in agreement with those in the literature and confirm the beneficial effect in hyperglycemic states of Physalis extracts. Accordingly, the fruits and aerial part of *Physalys alkekengi* L. have demonstrated a strong antidiabetic effect by inhibition of α-glucosidase activity and increasing of glucose transporter (GLUT) 4 expressions and insulin sensitivity in pre-adipocyte cells and HepG2-GFP-CYP2E1 cells [44]. In different models of diabetes in vivo, the ethyl acetate extracts prepared from aerial parts and fruits of *Physalys alkekengi* increased the uptake of glucose in HepG2 cells and reduced the glycemia, cholesterol, triglyceride and glycated protein levels and increased insulin sensitivity [45]. Qiu et al [40] also demonstrated an inhibitor effect of flavonoids from *Physalis alkekengi var. francheti* on nitric oxide production. The antioxidant activity has been demonstrated both in fruits and in stems of *Physalis alkekengi* and was due to the radical scavenger ability and lipid peroxidation inhibition [46,47]. It is known that hyperglycemia is associated with a cascade of events which finally leads to endothelial disfunction, proinflammatory state, oxidative stress, atherosclerosis, alteration of insulin sensitivity and obesity. The vascular microlesions occur in the endothelium due to metabolic or immunological factors, which induce the adhesion of platelets, monocytes and T lymphocytes, a process aggravated by the imbalance of vasodilator and vasoconstrictive factors [13]. In addition, the generation of advanced glycation end products causes the release of inflammatory mediators such as TGF-β and TNF-α and alters the gene transcription for some inflammation markers [12]. The secretion of pro-inflammatory cytokines stimulates the production of reactive oxygen species and/or reactive nitrogen species [48], which in turn intensifies the inflammation by activating some stress-activated kinases as well as stimulates transcription factors such as NFkB and activator protein-1 (AP-1) [49]. Accordingly, these factors upregulate the pro-inflammatory cytokine expression and amplify the inflammatory reaction. In our study, hyperglycemia was associated with lipid peroxides formation and secretion of proinflammatory cytokines, particularly IL-6, IL-31 and IL-33. In addition, hyperglycemia activated NFkB, maintained low levels of IL-10 and induced apoptosis. Therefore, targeting oxidative stress and inflammation can be an attractive strategy for prevention and control of hyperglycemic states.

NFkB is a key transcription factor for genes involved in survival, cell differentiation, inflammation and growth. In unstimulated cells, the inhibitory proteins family (IkB) keeps the NFkB sequestered in the cytoplasm. Inflammatory signals such as TNF-α or lipopolysaccharides induce phosphorylation of IκB proteins and its degradation, and consequently NFkB can enter the nucleus where it activates the genes involved in proliferation, cell transformation and angiogenesis [18]. In our study, the NFkB/pNFkB ratio increased in hyperglycemia compared to normoglycemia, suggesting the activation of the NFkB transcription factor in hyperglycemic conditions, in correlation with the increase of oxidative stress and inflammatory markers, especially IL-6, IL-31 and IL-33. In addition, oxidative stress and inflammation induced caspase-3 activation and cell death by apoptosis. It is known that the NFkB binding site is located on the IL-6 gene and this facilitates the IL-6 induction [50] by involvement of several factors such as p38 MAPK in neonatal rat cardiomyocytes [51] or TNF-α and oxidative stress in monocytes [52,53]. In turn, the upregulation of IL-6 is associated with significant induction of IL-1, TNF-α, macrophage chemoattractant protein-1 (MCP-1). Moreover, the high levels of IL-6 in plasma positively corelate with insulin resistance [54] and are explained by different mechanisms: the suppression of lipoprotein lipase and reduction of triglycerides levels in plasma [55], the activation of suppressor cytokine signaling proteins (SOCS) and blocking of STAT5B-mediated of the transcription factor activation for the insulin receptor [56,57]. The bidirectional crosstalk between oxidative stress, inflammation, the NFkB pathway and insulin resistance in the hyperglycemic state is complex and highlights its influence on endothelial dysfunction in diabetes.

Our extracts, especially PhyF, exerted protective effects on oxidative stress and inflammation, increased IL-10 secretion and diminished caspase-3 active form. An interesting effect of PhyF and quercetin is on IL-10, a cytokine known for anti-inflammatory and immunoregulation effects [58]. IL-10 inhibited the cytokines synthesis (IL-6 and TNF-α) and their effect on target cells [26]. Moreover, IL-10 can activate in cells the signal transducer and activator of the transcription 3 (STAT3) factor and the transcription of genes dependent on STAT3 such as SOCS3 [59]. In our study, both doses of extract and quercetin increased significantly the IL-10 levels, thus demonstrating the protective effect against inflammation in HUVECs exposed to hyperglycemia. In diabetes, the beneficial role of IL-10 on pancreatic β-cell functions and skeletal muscle was demonstrated in diabetes type I [60]. Thus, IL-10 increased pancreatic β-cell functions in response to glucose in vitro, increased insulin sensitivity, protected the skeletal muscle against macrophage infiltration, and reduced the side effects of proinflammatory cytokines on insulin signaling and glucose metabolism [28]. Furthermore, the administration of IL-10 improved the insulin resistance after lipid infusion [60], increased the glucose uptake in isolated adipocytes and diminished the insulin resistance induced by TNF-α [61].

A special mention is related to IL-33, cytokine with a dual role in diabetes, either as a proinflammatory and anti-inflammatory agent, dependent on the experimental model used. Thus, IL-33 had a protective effect against obesity, insulin resistance and diabetes in animal models [62] due to the reduction of resistin expression, accumulation of Th2 cells and IL-5, IL-13 and the suppression of T effectors cells by IL-10 releasing [63]. Additionally, IL-33 reduced the lipid storage and changed the macrophages phenotype towards protective one [64]. In other studies, IL-33 functioned as an alarm for the immune system in cellular necrosis and stress and induced the activation of NFkB, MAPK and the release of Th2 cytokines. In our study, hyperglycemia was associated with high levels of IL-33 in cell lysates, the effects being attenuated by quercetin, both doses of PhyH and low doses of PhyF. In normoglycemia, IL-33 acts as protective nuclear factor which reduces NFkB-triggered gene expression and thus dampens proinflammatory signaling [21]. The highly dependence of IL-33 functionality on cellular medium and also on normal or pathologic conditions may explain its different behavior in normo- and hyperglycemia.

The same pattern was noticed in normoglycemia regarding the effect of two extracts and quercetin on lipid peroxidation and TNF-α secretion. Thus, PhyH, PhyF and quercetin increased MDA and TNF-α levels in normoglycemia while in hyperglycemia they exerted protective effects against oxidative stress and inflammation. The contradictory results can be explained also by dual action of the extracts. Numerous studies indicate that natural extracts can exert different activity depend on the dose used or physiologic/pathologic context which characterize the biological system where they act. Fukumoto and Mazza [65] noted dual antioxidant and pro-oxidant activities for some polyphenols such as gallic acid, protocatechuic acid, syringic acid, vanillic acid, ellagic acid, caffeic acid, coumaric acid, chlorogenic acid, ferulic acid, myricetin, quercetin, rutin, kaempferol, (+)-catechin, (−)-epicatechin, delphinidin and malvidin. Generally, the polyphenols exert antioxidant effect in low dose or can act as prooxidants and produce free radicals, causing DNA damage and mutagenesis, in high dose. In addition, the prooxidant activity is due to metals found in the biological systems, particularly transition metals such as Fe and Cu. Instability of the polyphenols at alkaline pH; the composition of the cell culture medium in inorganic salts, vitamins and amino acids; or depletion of intracellular glutathione are other factors that contribute to prooxidant activity of natural extracts [65,66].

IL-31 belongs to the IL-6 cytokine family and is secreted in leukocytes after IL-33 stimulation. IL-31 contributes to the releasing of various proinflammatory cytokines and the inhibition of its secretion reduces the inflammation. Our results confirm IL-31 secretion in the hyperglycemic state and its inhibition after treatment with both doses of Physalis extracts and quercetin, demonstrating thus their beneficial effects on inflammation associated with hyperglycemia. Moreover, the inhibition of cytokines production by Physalis fruits extract inhibited the caspase-3 activation and consequently the cell death by apoptosis.

It is known that apoptosis involves two main pathways: the extrinsic or death receptor pathway and the intrinsic or mitochondrial pathway [17]. Both pathways lead to DNA fragmentation, the alteration of cytoskeleton and finally the formation of apoptotic bodies. It is known that, in HUVECs, hyperglycemia initiates apoptosis by intrinsic signals [67], which involves the release from the mitochondria of apoptogenic factors such as cytochrome c and leads to the caspase’s activation, morphological and biochemical changes and cell death [68]. Growth factors, DNA damage, intracellular Ca^2+^ and oxidative stress are the main triggers for the intrinsic pathway [69,70]. They induce upregulation of mitochondrial Bax and the downregulation of hexokinase 2 and Bcl-2 [71] and determine the cell death. ROS generation in hyperglycemia due to protein glycation, protein kinase C activation or mitochondrial oxidative phosphorylation is closely related to apoptosis and the inhibition of their production under PhyF exposure reduces caspase-3 active form and apoptosis and promotes cell recovery.

## 4. Materials and Methods

### 4.1. Preparation of Two Physalis Extracts for Lyophilization

The *Physalis alkekengi var. franchetii* L. were collected between June and August 2018 (Bihor County). A specimen of the species was kept in the Herbarium of Pharmacy Department, University of Oradea (registered in NYBG Steer Herbarium, code: UOP 05123). The fruits were crushed and used fresh to obtain the extract and the plant was dried at 22 ± 2 °C, on trays kept in a well-ventilated room. Physalis dried herba and Physalis fruits were subjected to maceration with a hydroalcoholic mixture (ethyl alcohol:distilled water 70:30). For the extraction, the ratio of vegetable product-hydroalcoholic mixture was 1:5. Extraction was carried out at a temperature of 22 ± 2 °C for 6 days. At the end of the extraction time, we separated the extractive residue solutions [72,73], and the extracts obtained were used for the subsequent determinations.

The hydro-alcoholic extracts were brought to rotavapor to remove the alcoholic fraction. The aqueous fraction of the two extracts was subjected to lyophilization (−85 °C) using a high-performance drying system as a laboratory lyophilizer.

### 4.2. Identification of the Polyphenols and Flavonoids Compounds of Two Physalis Extracts

Identification of phenol acids in extracts of Physalis herba compared to the extract of Physalis fruits was performed by HPLC using an Agilent 1200 HPLC system equipped with a diode array detector (PDA) (Santa Clara, CA, USA). A Zorbax SB-C18, 250x4, 6 mm, 100 A (S/No86996-11, B/No: 5701-029) column (Santa Clara, USA) was used. The mobile phase used for elution consisted of distilled water–formic acid (99.9:0.1) (*v*/*v*) (A) and acetonitrile (B). The acquisition parameters used were flow rate 1.5 mL/min, injection volume 10 μL, column temperature 25 °C and detection was performed at multiple wavelengths: 230, 260, 290, 300 and 360 nm. The polyphenols from extracts were identified by comparing the retention times from extracts chromatograms and a standard solution chromatogram.

The standard solution was prepared by mixing 1 mL of stock standard solutions of gallic acid, cinnamic acid, caffeic acid, ferulic acid, syringic acid, catechin, quercetin, rutin (flavonoid) and resveratrol (1000 ppm) and 1 mL of chlorogenic acid stock standard solution (500 ppm) and was injected in triplicate. All standards were from Sigma Aldrich (Darmstadt, Germany). The stock standard solutions and the extracts were prepared using a solvent ethanol 30%.

The total polyphenols content expressed in mg GAE/100 g DW (mg gallic acid equivalent/100 g dry weight) was determined by using the Folin–Ciocâlteu method, and total flavonoid content in mg QE/mL (mg quercetin/100 g DW) was evaluated by a colorimetric method.

#### 4.2.1. Phenolic Determination (Folin–Ciocâlteu Method)

The estimation of the total phenolic content was determined according to the Folin–Ciocâlteu method following Vicaș et al., with some modifications. Total phenolics are calculated as GAE using the regression equation based on the calibration curve: y = 1.9735x + 0.0261 (R^2^ = 0.9928), where x is the absorbance and y is the gallic acid equivalent (mg/mL) [74,75].

#### 4.2.2. Determination of Total Flavonoids

The total flavonoids content expressed as mg quercetin equivalent QE/100 g was evaluated using a colorimetric method developed by Kim et al. (2003). The samples were read against a prepared water blank at 510 nm. The equation based on the calibration curve was y = 56.818571x − 0.066498 (R^2^ = 0.9983), where x represents the absorbance and y is the mg quercetin [76].

### 4.3. Evaluation of Antioxidant Capacity

The antioxidant activity of the two Physalis extracts was estimated by DPPH (1,1-diphenyl-2-pycrylhydrazyl), FRAP (Ferric reducing antioxidant power) and CUPRAC (Cupric ions Cu^2+^ reducing), used previously to describe the pharmacological properties of other natural extracts.

#### 4.3.1. DPPH Method

The DPPH method was used to test the ability of compounds to act as free radical scavengers or hydrogen donors and evaluate the antioxidant capacity using a slightly modified method of Brand Williams et al. [77]. The color change (from dark violet to light yellow) correlated with the antioxidant capacity was performed at 517 nm on a Shimadzu UV-VIS spectrophotometer (Almere, The Netherlands). The free DPPH radical inhibition percentage was determined using the following formula: percent inhibition = [(AB − AA)/AB] × 100, where AB is the absorption of blank sample (t = 0 min), AA is the absorption of test extract solution (after 15 min).

#### 4.3.2. Ferric Reducing Antioxidant Power (FRAP) Method

The FRAP method evaluates the antioxidant capacity of a natural extract and is based on the change in color from light yellowish-green to blue of ferric tripyridyltriazine complex (Fe(III)TPTZ) and on the reduction of the ferric ion to the ferrous iron (Fe(II)) by a reductant, at an acid pH [78]. The calibration curve was made for concentrations between 0 and 300 μM, having a correlation coefficient R^2^ = 0.9956 and the regression equation (y = 0.0017x + 0.0848), where y represents absorbance performed at 595 nm. The antioxidant capacity was expressed as μmol Trolox equivalents (TE)/100 g dry weight (DW).

#### 4.3.3. Cupric Ions (Cu^2+^) Reducing-CUPRAC Assay

The CUPRAC method was used to determine the antioxidant activity of the extract. This method was evaluated by the cupric ions (Cu^2+^) reducing capacity based on the change in color from light green to reddish-orange of a complex with copper of neocupreine, 2,9-dimethyl-1,10-phenantroline. The spectrophotometric determination was performed at 450 nm after 30 min, and Trolox was used as a standard solution. Increased absorbance of the reaction mixture indicates high reduction capability. The calibration curve was made for concentrations between 0 and 500 μM, and the results were expressed as μmol Trolox equivalent/g DW.

### 4.4. In Vitro Experiments

#### 4.4.1. Cell Cultures

The assays were performed on the commercial human umbilical vein endothelial cells (HUVEC) obtained from the European Collection of Cell Cultures (ECACC, Porton Down, Salisbury, UK). The cells were multiplied in RPMI medium, supplemented with 10% fetal calf serum (FCS), antibiotics and anti-mycotic (Biochrom AG, Berlin, Germany) in a humidified CO_2_ incubator at 37 °C. The surfaces markers of cells, ICAM-1, CD29, CD34, CD73, CD90 and CD105, were analyzed using flow cytometry as previously published [79]. Cell cultures in the 23rd to 26th passages were used. All reagents were purchased from Sigma Aldrich, Co (Heidelberg, Germany).

#### 4.4.2. Viability Assay

The cells survival under exposure to the Physalis dried herba (PhyH) and Physalis fruits (PhyF) were tested through the colorimetric measurement of formazan using CellTiter 96^®^ AQueous Non-Radioactive Cell Proliferation Assay (Promega Corporation, Madison, WI, USA). Human umbilical vein endothelial cells (HUVECs) were cultivated at a density of 10^4^/well in 96-well plates (TPP, Trasadingen, Switzerland), for 24 h and then treated for 24 h with different concentrations of PhyF and PhyH. To make the necessary dilutions for cell treatment, two extract solutions were prepared as follows: PhyF 3.13 mg DW/mL containing 33.970 µg GAE/mL and PhyH 20.3 mg DW/mL containing 45.101 µg GAE/mL. The solutions were further diluted with cell growth medium to obtain the dilutions (ranged 0.0004–20 µg GAE/mL) for both extracts. The optical density values were read at an absorbance of 540 nm using an ELISA plate reader (Tecan, Männedorf, Switzerland) for MTS. All the experiments were conducted in triplicate. Untreated cultures exposed to medium were used as controls. Results are presented as percent of untreated controls. The doses that decreased viability levels below 70% were considered toxic.

#### 4.4.3. Cell Lysates

The HUVECs cells, seeded on Petri dishes at a density of 10^4^/cm^2^, were exposed for 24 h to 0.01 µ/mL quercetin and to two doses of Physalis extracts, 0.1 µg GAE/mL and 0.05 µg GAE/mL for 24 h. The two doses of Physalis extracts were chosen based on viability tests, below values that decrease the cell viability. Untreated cells were used as controls. Afterwards, the cells were washed 3 times with PBS and incubated for 24 h either in normoglycemia (2.12 mM) or hyperglycemia (30 mM). The level of glucose was chosen based on literature data which studied the endothelial dysfunction in normal and abnormal glucose metabolism [80,81]. Cells were collected by scraping and treated as previously described [79]. The protein level was determined by the Bradford method (Biorad, Hercules, CA, USA). All in vitro experiments were conducted in triplicate.

#### 4.4.4. Oxidative Stress, Inflammation Markers and Transcription Factors Assessment

Oxidative stress was quantified by the evaluation of malondialdehyde levels in cell lysates through the fluorimetry with 2-thiobarbituric acid, as described by Conti et al. [82]. The MDA was spectrofluorimetrically evaluated in the organic phase using a synchronous technique with excitation at 534 nm and emission at 548 nm, and the results were expressed as nmoles/mg protein. Inflammation was assessed after quercetin and both extract treatments by the measurement of IL-31 and IL-33 levels in cell lysates using ELISA immunoassay kits from R&D Systems (Minneapolis, MN, USA), and the results were expressed as pg/mL. Then, based on these results, the NFkB, phosphorylated NFkB (pNFkB), IL-6 and IL-10 expressions in cell lysates, TNF-α secretion (mTNF-α) and caspase-3 active form in supernates after exposure to only PhyF were measured. NFkB, pNFkB, IL-6 and IL-10 were evaluated by Western blot analysis and TNF-α and the active caspase-3 by ELISA using Quantikine^®^ caspase-3 ELISA kit (R&D Systems Inc., Minneapolis, MN, USA). For Western blot, 20 µg protein/lane were separated by electrophoresis on SDS PAGE gels and then transferred to polyvinylidenedifluoride membranes (Biorad Miniprotean system from BioRad) as previously published [83]. Blots were blocked and then incubated with antibodies against NFkB, pNFkB, IL-6, IL-10 and GAPDH and then further washed and incubated with corresponding secondary peroxidase-linked antibodies (Santa Cruz Biotechnology, Dallas, TX, USA). The proteins were detected using Supersignal West Femto Chemiluminiscent substrate (Thermo Fisher Scientific, Rockford, IL, USA), and then were quantified using Quantity One analysis software (Biorad). Image analysis of Western blot bands was done by densitometry; the results were normalized to GAPDH.

#### 4.4.5. Statistical Analysis

The statistical significance of the results was conducted by using one-way ANOVA followed by Tukey’s Multiple test. All reported data were expressed as the mean of triplicate measurements ± standard deviation (SD) and a *p* value lower than 0.05 was considered statistically significant.

## 5. Conclusions

In the present study, we bring important knowledge regarding the interaction of endothelial cells, hyperglycemia and two doses of Physalis puree extracts and quercetin, in order to simulate the pathogenetic mechanisms involved in the endothelial dysfunction in hyperglycemia. The results demonstrate that Physalis extracts, especially prepared from fruits or PhyF, had an important content of phenolic compounds with a high antioxidant activity. The Physalis extracts and quercetin inhibited the lipid peroxidation and diminished the secretion of proinflammatory cytokines, and consequently inhibited the apoptosis induced by hyperglycemia, particularly the extract prepared from fruits. These experimental findings suggest that *Physalis alkekengi var. franchetii* L. may be a new therapeutic approach to decrease the oxidative stress, inflammation and apoptosis associated with hyperglycemia in HUVECs. However, further studies with multiple doses and exposure times are necessary to demonstrate the real clinical efficiency in diabetes.

## Figures and Tables

**Table 1:** Content of flavonoids and phenolic acids determined in Physalis extracts.

Bioactive Compounds	λ_max_ (nm)	R_T_ (min)	Physalis Fruits mg/kg *	Physalis Herba mg/kg *
(±) Catechin	230	10.35	1.698 ± 0.25	-
Rutin	260	13.56	14.505 ± 0.32	0.816 ± 0.45
Syringic acid	230	11.62	3.416 ± 0.41	0.924 ± 0.65
Caffeic acid	300	11.63	0.761 ± 0.15	0.622 ± 0.35
Chlorogenic acid	300	14.94	45.20 ± 0.47	-
Ferulic acid	300	14.58	5.719 ± 0.56	0.307 ± 0.82
Gallic acid	230	4.98	32.697 ± 0.97	31.025 ± 0.28
Resveratrol	300	18.24	22.626 ± 0.31	-

* Results are expressed as a mean ± standard deviation.

**Table 2:** The total polyphenols, flavonoids and the antioxidant capacity of Physalis extracts.

No.	Methods	Physalis Fruits Extract *	Physalis Herba Extract *
1	Total Polyphenols mg GAE/100 g DW	1082.5 ± 1.98	221.6 ± 1.68
2	Flavonoids mg QE/100 g DW	0.695 ± 0.53	0.297 ± 0.53
3	DPPH %	75.00 ± 0.29	30.45 ± 0.31
4	FRAP µmol TE/100 g DW	489.71 ± 1.02	111.18 ± 0.87
5	CUPRAC µmol TE/100 g DW	327 ± 1.95	99.15 ± 1.21

* Results are expressed as a mean ± standard deviation.
